# Comparison of US County-Level Public Health Performance Rankings With County Cluster and National Rankings: Assessment Based on Prevalence Rates of Smoking and Obesity and Motor Vehicle Crash Death Rates

**DOI:** 10.1001/jamanetworkopen.2018.6816

**Published:** 2019-01-04

**Authors:** Megan Wallace, Joshua M. Sharfstein, Joshua Kaminsky, Justin Lessler

**Affiliations:** 1 Department of Epidemiology, Johns Hopkins Bloomberg School of Public Health, Baltimore, Maryland; 2 Department of Health Policy and Management, Johns Hopkins Bloomberg School of Public Health, Baltimore, Maryland

## Abstract

**Importance:**

Health departments can be grouped together based on sociodemographic characteristics of the population served. Comparisons within these groups can then help with monitoring and improving the health of their populations.

**Objective:**

To compare county-level percentile rankings on outcomes of smoking, motor vehicle crash deaths, and obesity within sociodemographic peer clusters vs nationwide rankings.

**Design, Setting, and Participants:**

This cross-sectional, population-based study of demographic and health data from the 2014 Behavioral Risk Factor Surveillance System and the 2016 Robert Wood Johnson Foundation County Health Rankings data set was conducted at 3139 of 3143 US counties and county-equivalents. Four locations were excluded due to incomplete data. Data analysis was conducted between January and August 2017.

**Exposures:**

Random forest algorithms were used to identify sociodemographic characteristics most associated with the outcomes of interest. These characteristics were race and ethnicity, educational attainment, age, marital status, employment status, sex, and health insurance status. *k*-means clustering was used to cluster counties based on these sociodemographic characteristics and the percentage of the county classified as rural.

**Main Outcomes and Measures:**

County-level smoking prevalence, motor vehicle crash death rate, and obesity prevalence. County percentile rankings on the outcomes of interest were compared in the national context and the within-cluster context.

**Results:**

A total of 318 856 967 individuals (mean [SD] individuals per county, 101 579.2 [326 315]; 161 911 910 women [50.8%]) were represented by the 3139 counties used in this analysis. Eight distinct sociodemographic clusters throughout the United States were found. Cluster-specific percentile rankings for both smoking prevalence and motor vehicle crash death rates improved more than 70 percentile points for several counties in the rural, American Indian cluster compared with the nationwide percentiles. Conversely, the young, urban, middle to high socioeconomic status cluster included counties with cluster-specific percentile rankings that declined by 60 percentile points or more compared with the nationwide rankings for all 3 outcomes of interest.

**Conclusions and Relevance:**

Comparing county health outcomes on a nationwide or statewide basis fails to adequately account for sociodemographic context. Clustering counties by sociodemographic factors related to the outcome of interest allows a better understanding of other factors that may be shaping the prevalence of health outcomes. These groupings may also aid learning exchange.

## Key Points

**Question:**

Can effective comparison groups be created to assess county-level public health performance?

**Findings:**

In this cross-sectional study including more than 300 million individuals in the United States, 8 groups of counties with similar sociodemographic characteristics were identified. Percentile ranks for outcome prevalence often differed when counties were considered within their cluster in comparison with a nationwide scale.

**Meaning:**

Supplying counties with peer groups created using sociodemographic similarities may help local health departments to compare progress and share techniques in efforts to address leading public health challenges.

## Teaser

This cross-sectional study compares US county-level percentile rankings on outcomes of smoking, motor vehicle crash deaths, and obesity within sociodemographic peer clusters vs nationwide rankings.

## Introduction

In 2015 and 2016, life expectancy in the United States declined for the first time in decades.^1,2^ There has been much discussion about the underlying cause of this trend shift, but it is clear that, overall, America’s health is no longer improving. In addition, there are great disparities in health outcomes across the United States, with individuals of high socioeconomic status (SES) continuing to have better health outcomes, including longer life expectancy, than those of low SES.^3,4^ These gaps appear to be widening. When considered on a county level, those counties with the highest life expectancies have continued to increase life expectancy over the past 25 years, while those with the lowest life expectancies have plateaued.^4,5^

Local health departments are on the front lines of this battle to improve health in the United States, often with few resources and data to judge their performance.^6,7,8^ Local health departments often look to metrics such as the Robert Wood Johnson County Health Rankings as a means to evaluate their performance in relation to their same-state peers.^9^ However, these comparisons frequently reflect the underlying socioeconomic differences within a state, rather than the effectiveness of local health programs. Comparisons of different populations within a state may obscure net gains being made by health departments serving low SES populations. In some cases, health departments working with hard-to-serve populations may even be harmed in competitive funding situations. Population interventions are often specialized to the target population to improve effectiveness; therefore, if a county ranking last in a state were to implement all of the same interventions as the county that ranked first, it is not certain that the interventions would have the same success.^10^

There is a need for appropriate comparison groups that health departments can use for benchmarking as a forum for learning exchange. This is not a new concept; urban health departments have been reaching out to one another for years, the Big Cities Health Coalition being a prime example.^11^ This group has a data platform for comparisons between the largest cities on key health indicators and provides case studies on interventions by urban health departments. However, this leaves a void for rural health departments, which often lack formal relationships across state lines, and have seen stagnating health improvement as compared with their urban counterparts.^12,13^ The Centers for Disease Control and Prevention (CDC) launched peer groups as part of their Community Health Status Indicators Project in 2008.^14,15^ These groups have since been updated in a collaboration with the county health rankings using methods modeled after the Canadian health region peer groups.^16,17^ While these peer groups begin to provide local health departments with the comparisons they need, it is unclear how the social determinants used to group counties were selected. Therefore, these metrics may not be the most important for the outcomes of interest. Clustering based on sociodemographic characteristics most important for the outcomes of interest creates groupings that may engage in learning exchange more effectively because these counties will be introducing interventions in populations that share characteristics known to be meaningful for the target outcomes. Use of the CDC methods also creates many smaller county groupings, which limits the number of comparison counites within each group. Here we create statistically derived comparison groups for local health departments based on the sociodemographic makeup of the county populations and evaluate the distributions of important health behaviors within the comparison groups.

## Methods

### Study Data

The study’s analysis used individual-level data from the 2014 Behavioral Risk Factor Surveillance System and county-level data from the 2016 Robert Wood Johnson Foundation County Health Rankings data set, with additional measures included from the US Census Bureau American Community Survey (eTable 1 in the Supplement).^18,19,20^ These data sets are deidentified and publicly available. The Johns Hopkins Bloomberg School of Public Health institutional review board office determined this study was not human subjects research and did not require institutional review board oversight. Analysis took place between January and August 2017. This article is compliant with the Strengthening the Reporting of Observational Studies in Epidemiology (STROBE) reporting guideline for cross-sectional studies.

### Statistical Analysis

To evaluate local public health performance in this study, we chose to focus on the CDC Winnable Battles,^21^ which are priorities with large-scale health effects and known effective strategies to address them. Specifically, due to data availability, we evaluated county-level smoking prevalence, obesity prevalence, and motor vehicle crash mortality across the United States.

To identify the individual characteristics that were most predictive of outcomes of interest, we used the random forest algorithm to rank the importance of variables present in the individual-level data from the 2014 Behavioral Risk Factor Surveillance System in assuming these outcomes.^22,23^ This analysis was done using the randomForest package in the R statistical language (R Foundation for Statistical Computing). We accounted for unbalanced outcome distributions using stratified sampling by the outcomes of interest. The 2014 Behavioral Risk Factor Surveillance System sampling weights were not used. Observations with missing data were eliminated from the analysis. Self-reported seatbelt use and driving under the influence of alcohol were used to evaluate the individual characteristics most associated with motor vehicle crash mortality, because these outcomes are on the pathway to motor vehicle crash death.^24^ The mean decrease in the probable accuracy, which is the normalized average difference between the prediction error before and after permutating each predictor variable, was used to determine which predictor variables would be included in further analyses. Variables whose removal led to a mean decrease in accuracy of 10% or higher were considered associated with the outcomes of interest. Most variables tested met this threshold for at least 1 outcome of interest. These variables were individual race and ethnicity, educational attainment, age, marital status, employment status, sex, and health insurance status. Variables that did not meet the threshold were internet access and home ownership. All variables were categorized according to the classifications used in the original data sets.

Proxies for these variables available at the county level were used to create county clusters. These variables were non-Hispanic black, non-Hispanic white, Hispanic, American Indian, Asian, some college education, median age, married, unemployed, female, and uninsured. No measure of population density was available in the individual-level data, but due to its relationships with the outcomes and potential impact on intervention effectiveness, percent of county classified as rural was also included in further analysis.

We used *k*-means analysis to identify clusters of counties with similar sociodemographic profiles. The sociodemographic characteristics of each of the clusters were investigated, and clusters were mapped for visualization of the location of the clusters across the United States.

Counties were first given overall percentile ranks for each outcome based on their current smoking prevalence, obesity prevalence, and motor vehicle crash death rates, compared with all other counties in the United States. Outcome percentile ranks were then calculated separately within cluster, comparing a county’s outcome prevalence only with other counties within its cluster. These cluster-specific outcome percentiles were compared with the previous nationwide percentiles. Counties missing data for any of the outcomes of interest were excluded from percentile comparisons for that outcome. These nationwide and cluster-specific county percentiles were mapped to highlight the high- and low-percentile counties before and after clustering.^25^ Cluster-specific means, variances, and average percentile change after clustering were calculated for each outcome. Data were analyzed using R, version 3.2.2.^26^

## Results

**Figure 1.**
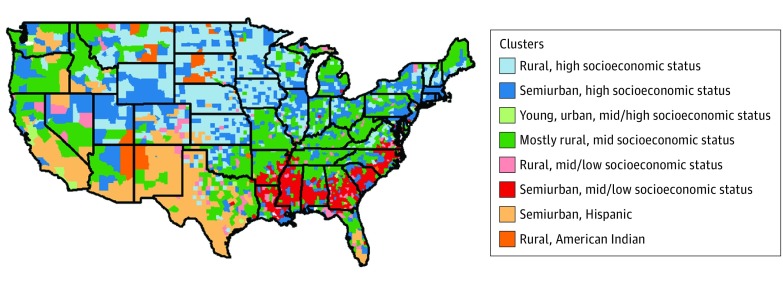
Map of the Sociodemographic-Based County Clusters

The sociodemographic characteristics of each cluster of counties can be found in eTable 2 in the Supplement. Gap statistics, Bayesian information criterion, and within-group sum of squares were used to select the optimal number of clusters.^27,28,29^ These methods supported from 6 to 10 clusters, and we chose to base our analysis on the median number of 8 clusters. The clusters vary markedly, particularly in racial and ethnic breakdowns, socioeconomic markers, and the percentage of the counties classified as rural. We assigned each cluster a name that highlights the makeup of its population. The 8 clusters identified were rural, high SES; semiurban, high SES; young, urban, middle to high SES; mostly rural, middle SES; rural, middle to low SES; semiurban, middle to low SES; semiurban, Hispanic; and rural, American Indian. Figure 1 displays the locations of the clusters, which tend to group together geographically.

The prevalence of the outcomes varied between clusters. The nationwide mean (SD) county-level smoking prevalence was 18.4% (3.8%). The young, urban, high to middle SES group had the lowest mean (SD) percent of current smokers at 12.9% (2.6%), while the rural, American Indian group had the highest at 29.3% (6.2%) (Table). The nationwide mean (SD) county-level motor vehicle crash death rate was 19.7 (9.6) per 100 000 population. Motor vehicle crash death rates ranged from a mean (SD) of 7.2 (3.2) per 100 000 population in the young, urban, middle to high SES group to 46.4 (16.8) per 100 000 population in the rural, American Indian group. The nationwide mean (SD) county-level obesity prevalence was 30.9% (4.5%). The young, urban, middle to high SES group also had the lowest obesity prevalence at 23.2% (4.3%), while the semiurban, middle to low SES group had the highest at 35.6% (3.9%).

**Table.** Cluster Variance and Average Percentile Change for Each Cluster by Outcome

Outcome	Rural, High SES (n = 674)	Semiurban, High SES (n = 727)	Young, Urban, Middle to High SES (n = 37)	Mostly Rural, Middle SES (n = 973)	Rural, Middle to Low SES (n = 116)	Semiurban, Middle to Low SES (n = 326)	Semiurban Hispanic (n = 244)	Rural, American Indian (n = 42)	Overall
Smoking									
Smoker, mean (SD), %	16.1 (1.9)	17.3 (3.1)	12.9 (2.6)	19.9 (3.5)	20.4 (3.8)	21.3 (2.8)	16.3 (2.1)	29.3 (6.2)	18.4 (3.8)
Mean percentile change^a^	19.6	8.0	38.4	–12.7	–17.4	–25.2	16.4	–43.9	NA
Motor vehicle crash deaths									
Motor vehicle death rate (per 100 000 population), mean (SD), %	20.4 (9.7)	12.2 (4.9)	7.2 (3.2)	23.0 (8.0)	23.8 (7.3)	23.7 (9.0)	20.4 (9.1)	46.4 (16.8)	19.7 (9.6)
Mean percentile change^a^	–2.6	26.2	41.8	–12.8	–15.7	–13.1	–2.9	–39.8	NA
Obesity									
Obesity, mean (SD), %	30.1 (3.5)	29.0 (4.3)	23.2 (4.3)	31.9 (3.6)	32.7 (4.2)	35.6 (3.9)	28.8 (4.2)	35.1 (4.2)	30.9 (4.5)
Mean percentile change^a^	7.2	13.4	35.4	–7.2	–12.3	–30.6	15.4	–28.5	NA

Abbreviations: NA, not available; SES, socioeconomic status.

^a^ Represents the mean percentile change from the nationwide percentiles to the cluster-specific percentiles.

**Figure 2.**
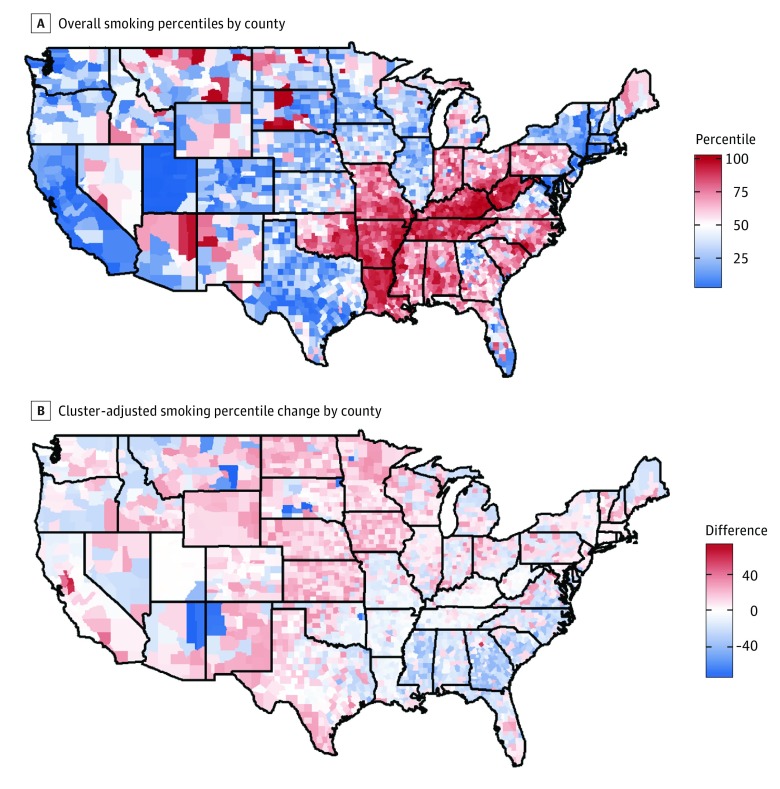
Map of Overall Smoking Percentile by County and Cluster-Adjusted Smoking Percentile Change by County

**Figure 3.**
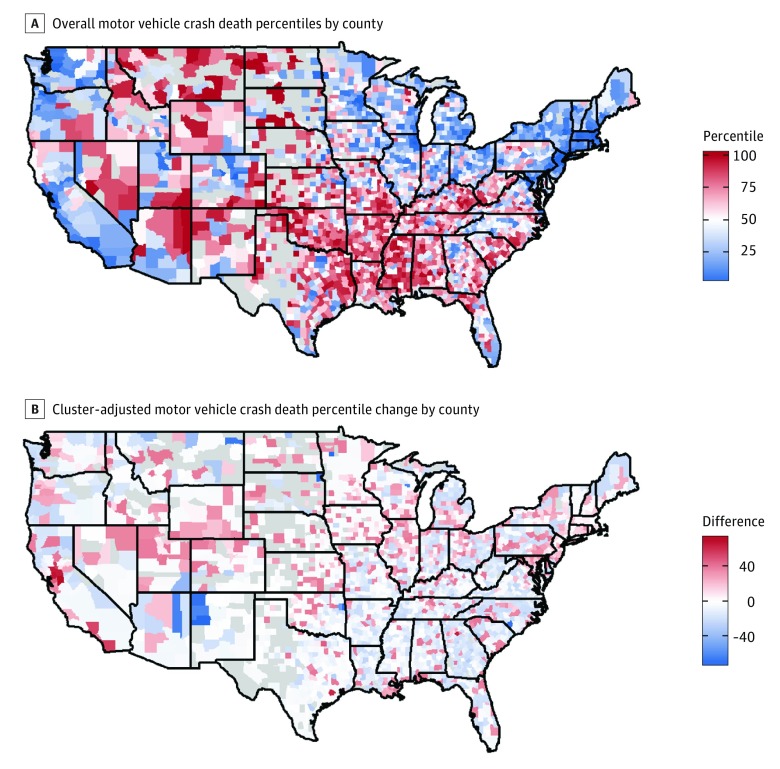
Map of Overall Motor Vehicle Crash Death Percentile by County and Cluster-Adjusted Motor Vehicle Crash Death Percentile Change by County

**Figure 4.**
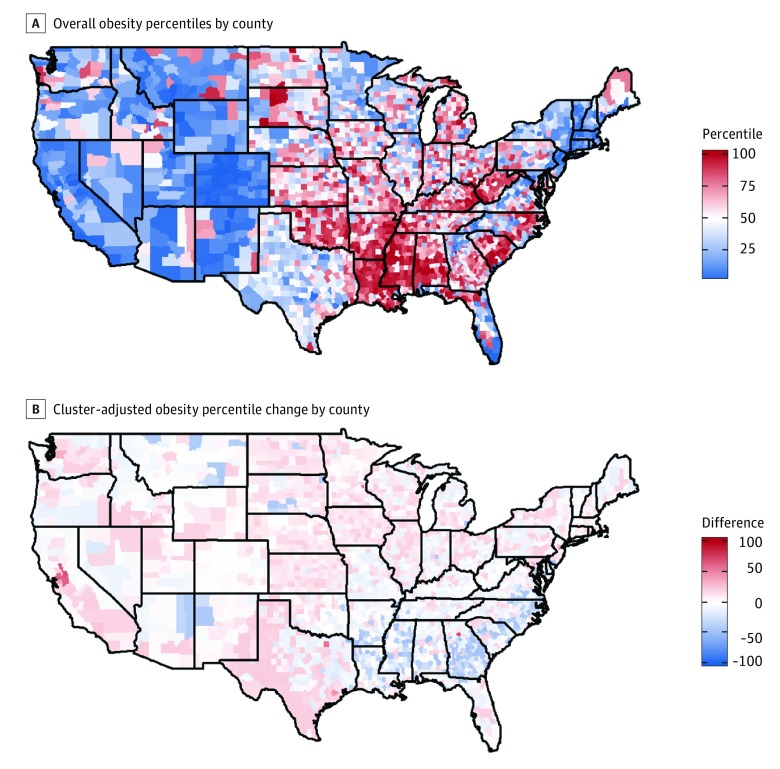
Map of Overall Obesity Percentile by County and Cluster-Adjusted Obesity Percentile Change by County

Nationwide county-level outcome percentile rankings often shifted when those percentiles were recalculated within clusters (Figure 2B, Figure 3B, and Figure 4B). Across all outcomes, the national percentile rankings showed regional trends, while the within-cluster percentiles had more geographical heterogeneity. Additionally, groups composed of mostly high-performing counties, ie, those with lower outcome percentiles, saw an increase in their average percentile after clustering, because many of the counties within the group had an increased percentile when considered within the context of their cluster. Likewise, groups composed of counties with worse outcomes, ie, those with higher outcome percentiles, tended to experience percentile decreases on average (Table).

As can be seen in Figure 2, smoking tends to be concentrated in the Mississippi Valley and Appalachian Highlands regions of the United States. However, after clustering, much of the Southeast showed improvements in their relative percentile, indicating that these areas are doing well compared with demographically similar counties, while some counties in the northern Midwest and Mountain regions have higher percentiles after clustering, suggesting that these counties have a high smoking prevalence compared with demographically similar counties. Several counties in the rural, American Indian group had within-cluster percentiles that were 70 points lower than their nationwide percentiles. For example, in Rosebud County, Montana, 23% of the population are current smokers, placing the county in the 88th percentile overall; however, it is in the 14th percentile compared with the demographically similar counties in its cluster. Conversely, Sacramento County, California, has a smoking prevalence of 15%, placing it in the 16th percentile nationwide, but in the 80th percentile within its cluster.

Figure 3 shows that motor vehicle crash death rates are the highest in the most rural, central regions of the United States, where driving distances tend to be longer. This pattern was sustained after cluster-specific percentiles were calculated; however, there were notable changes for individual counties. This outcome also had many counties within the rural, American Indian cluster that had within-cluster percentiles more than 70 points lower than their nationwide percentiles. Cibola County, New Mexico, had the largest difference; with a motor vehicle crash death rate of 27 per 100 000 population, the county was in the 81st percentile nationwide, but when considered within its cluster, it falls within the 8th percentile. The young, urban, high to middle SES group had low rates of motor vehicle crash deaths overall; thus, high-performing counties still show room for improvement compared with their cluster peers. For example, San Joaquin County, California, has a motor vehicle crash death rate of 12 per 100 000 population, placing it in the 21st percentile overall, but within its cluster in the 92nd percentile.

The highest obesity percentiles are concentrated in the Southeast and Appalachian Highlands regions (Figure 4A). However, after clustering the highest percentiles are no longer as thickly concentrated in these regions, and again show up frequently in the Midwest (Figure 4B). One regional high performer is Miller County, Arkansas, which has an obesity prevalence of 34%, placing it in the 73rd percentile nationwide, but within the semiurban, middle to low SES cluster in the 26th percentile. The young, urban, middle to high SES group had the lowest average obesity prevalence of any cluster, and within this group Sutter County, California, has an obesity prevalence of 28%, placing it in the 21st percentile overall, but within its cluster in the 89th percentile.

## Discussion

This method demonstrates that there are clear sociodemographic clusters of counties throughout the United States, and these clusters differ in the prevalence of 3 key outcomes related to the CDC Winnable Battles. Although the clusters did have regional tendencies, states had counties from many different clusters within their borders. This suggests that comparisons within states may not account for the heterogeneity found within states, and that counties may share more sociodemographic similarities with counties outside of their states.

There are other examples of county-level clustering schemes, such as the American Communities Project^30^ or Eight Americas.^31^ While Murray et al^31^ were focused on highlighting the disparities that exist between groups across the United States, the current study’s work focuses on comparing counties within similar groups. Due to this difference in focus, the methods used here differ from others by creating clusters based on sociodemographic characteristics most associated with the outcomes of interest in a reproducible algorithmic manner. This places an emphasis on not just creating clusters of similar counties but creating clusters that are similar in the sociodemographic variables most important for the outcomes of interest. Additionally, because the clustering in this article was based on sociodemographic indicators that are possibly associated with the outcomes of interest, one might hypothesize that members of a given cluster would benefit from similar public health interventions, making these clusters excellent groups for learning exchange.^30,31^ This method provides a tool for comparing county performance with other counties with similar populations. Those counties with the highest percentiles overall will continue to have the highest percentiles within their clusters; likewise those with the lowest percentiles overall will continue to have the lowest percentiles within their clusters. However, those counties that have moderate performance on a nationwide scale, but are in a cluster that performs well on the outcomes of interest, will have high within-cluster percentiles, as was seen in Sacramento County, California, San Joaquin County, California, and Sutton County, California. These high within-cluster percentiles indicate that, although they may be doing well in the context of the entire United States, other counties with similar sociodemographic makeups tend to have better performance, which signals an opportunity for further improvement in these counties. Additionally, counties with moderate performance that belong to low-performing clusters, had low within-cluster percentiles as was seen in Rosebud County, Montana, Cibola County, New Mexico, and Miller County, Arkansas. These low within-cluster percentiles suggest that although these counties have significant room for improvement in the national context, they are doing well for their respective groupings and should serve as examples for other counties in their clusters of how to make meaningful improvements.

The sociodemographic indicators that were associated with the outcomes of interest had substantial overlap, allowing us to create a single clustering scheme that could be applied to all 3 outcomes. This suggests that this method could be applied to many other outcomes to obtain similar rankings and comparisons with few, if any, adjustments. This would enable local health departments to compare their performance with other counties within their cluster for any outcome for which there are data available. Additionally, since these sociodemographic indicators on which the clusters are based appear to be consistently important, local health departments could engage in learning exchange, even for those outcomes for which there is little data available, to implement interventions that are likely to be the most effective within their populations.

The importance of the social inequities of health cannot be overemphasized, and evaluations that highlight these detrimental health gaps are vitally important, but the social inequities are often barriers that few local health departments have the resources to affect, particularly those health departments that have the worst health outcomes overall. This method provides a mechanism to create valuable comparison groups, through which the lowest performers within a cluster can gain valuable information on how to improve their performance by implementing similar interventions as the highest performers in the cluster.

This is not to say that counties that perform well for their given clusters should consider their work done. These high-performing counties should be looking to new and innovative ways to continue to improve the health of their populations and close the health gap caused by social inequities. Likewise, it is not to say that counties with worse cluster percentiles than national percentiles should be considered failures because they do poorly within their cluster; rather it provides comparators that they can look to in order to find ways to further improve in their positive results. These methods provide an opportunity for counties to compare their performance with and learn from counties facing similar challenges. However, the potential for appropriate learning and benchmarking only can be realized if counties have a platform to review these data and engage with their peers.

While this method provides an important mechanism to evaluate county performance, it should be considered a supplementation and not a replacement of current ranking methods. Beyond the ability to highlight the social inequities of health there are additional reasons why within-state comparisons are logical and needed. Counties within a given state are subject to the same state legislation and are served by the same state health department, making differences within a state a meaningful tool, particularly for state-level decision making.

### Limitations

This study had limitations. Data availability was a limitation in this study, one that is frequently faced by local health departments searching for stable and comparable county-level data. To obtain stable county-level estimates, we often had to combine data for multiple years, meaning that county estimates may not be representative of the current outcome prevalence within a county. This would be particularly important for counties that have experienced recent, rapid health improvements or declines or sociodemographic shifts within their populations. In addition, we were unable to account for within-county variability in either the sociodemographic predictor variables or the outcomes of interest. Counties with high levels of sociodemographic heterogeneity would likely benefit from clusters at the subcounty level; however, this would require more granular data than is currently available. This study is also subject to the limitations of the secondary data sets used, including sampling variability and measurement error. However, these data came from large, well-validated surveys, and these limitations have negligible impacts on the study results.

The county-level proxies used in the cluster analysis originated from different data sources and had some categorization differences than the individual-level data used in the sociodemographic variable selection. Variables associated at the individual level may not be associated at the population level; however, the reduction in variance across clusters suggests our methods are capturing at least some of the population-level association.

## Conclusions

Our results demonstrated that when counties are grouped based on sociodemographic characteristics related to outcomes of interest, the outcome prevalence rankings can vary from the rankings in the nationwide context. Grouping counties based on sociodemographic characteristics not only provides a new framework for comparisons, but also aids the exploration of other factors that may be influencing the prevalence of health outcomes and facilitates learning exchange between similar counties.

## Notes

Supplement. **eTable 1.** Sources of County-Level Data **eTable 2.** Sociodemographic Characteristics by Cluster
