# Supplementary material for: Comparison of US County-Level Public Health Performance Rankings With County Cluster and National Rankings: Assessment Based on Prevalence Rates of Smoking and Obesity and Motor Vehicle Crash Death Rates
Source: JAMA Netw Open. 2019 Jan 4;2(1):e186816. doi: 10.1001/jamanetworkopen.2018.6816 (PMC6324334; doi:10.1001/jamanetworkopen.2018.6816)
Supplement: Supplement. — eTable 1. Sources of County-Level Data eTable 2. Sociodemographic Characteristics by Cluster [file jamanetwopen-2-e186816-s001.pdf]

Supplementary Online Content

Wallace M, Sharfstein JM, Kaminsky J, Lessler J. Comparison of US county-level public health performance rankings with county cluster and national rankings: assessment based on prevalence rates of smoking and obesity and motor vehicle crash death rates. *JAMA Netw Open*. 2019;2(1):e186816. doi:10.1001/jamanetworkopen.2018.6816

eTable 1. Sources of County-Level Data
eTable 2. Sociodemographic Characteristics by Cluster

This supplementary material has been provided by the authors to give readers additional information about their work.

© 2019 Wallace M et al. *JAMA Network Open*.

eTable 1. Sources of County Level Data

Measure	Source	Year(s)
Percent of adult population that are current smokers	Behavioral Risk Factor Surveillance System	2014
Motor vehicle crash death rate per 100,000 population	Centers for Disease Control and Prevention's Wide-ranging Online Data for Epidemiologic Research (WONDER) Mortality Data	2007-2013
Percent of adult population with Body Mass Index ≥ 30	Centers for Disease Control and Prevention's Diabetes Interactive Atlas	2012
Percent of population under age 65 without health insurance	Small Area Health Insurance Estimates	2013
Percent of population aged 16 or greater that are unemployed but seeking work	Bureau of Labor Statistics	2014
Percent of adults aged 25 to 44 with some post-secondary education	American Community Survey	2010-2014
Percent of population that identify as non-Hispanic African American	Census Population Estimates	2014
Percent of population that identify as American Indian and Alaskan Native	Census Population Estimates	2014
Percent of population that identify as Hispanic	Census Population Estimates	2014
Percent of population that identify as Non-Hispanic white	Census Population Estimates	2014
Percent of population that are females	Census Population Estimates	2014
Percent of county that is considered rural	Census Population Estimates	2010
Median Age	American Community Survey	2010-2014
Percent of adult population that are married	American Community Survey	2010-2014

© 2019 Wallace M et al. *JAMA Network Open*.

eTable 2. Sociodemographic Characteristics by Cluster

Characteristics	Rural, High SES N=674	Semi-Urban, High SES n=727	Young, Urban, High/Mid SES n=37	Mostly Rural, Mid SES n=973	Rural, Mid/Low SES n=116	Semi-Urban, Mid/Low SES n=326	Semi-Urban Hispanic n=244	Rural, American Indian n=42
Total Population	12,726,446	152,196,153	44,405,450	33,779,765	1,904,737	26,426,795	46,559,629	857,992
Mean Percent White	92.0	79.6	42.2	86.5	67.5	49.9	45.3	30.6
Mean Percent Hispanic	3.9	7.7	21.0	5.1	10.5	4.4	46.8	5.2
Mean Percent African American	1.1	7.2	8.9	4.7	18.2	42.9	4.3	1.1
Mean Percent Asian	0.6	2.5	20.8	0.6	0.7	0.9	1.7	0.7
Mean Percent American Indian	1.3	1.2	2.2	1.7	2.0	0.7	2.1	59.6
Mean Percent Some College	64.0	66.1	68.9	49.5	39.8	49.2	48.8	50.1
Mean Percent Married	59.0	50.5	49.4	53.7	44.1	42.7	51.0	41.3
Mean Percent Unemployed	4.5	5.4	5.8	7.2	6.9	8.7	6.0	9.5
Mean Percent Female	49.8	50.5	49.6	50.3	42.2	51.3	49.3	49.0
Mean Percent Uninsured	14.3	13.9	15.9	19.2	20.0	19.6	25.1	23.8
Mean Percent Rural	79.9	25.0	10.5	73.0	75.3	56.5	40.6	83.1
Median Age	44.4	37.4	37.0	43.1	40.1	38.6	36.2	31.7

Abbreviation: SES, socioeconomic status

© 2019 Wallace M et al. *JAMA Network Open.*
